# Supplementary material for: Mechanisms governing the pioneering and redistribution capabilities of the non-classical pioneer PU.1
Source: Nat Commun. 2020 Jan 21;11:402. doi: 10.1038/s41467-019-13960-2 (PMC6972792; doi:10.1038/s41467-019-13960-2)
Supplement: Supplementary file 7 — Source data [file 41467_2019_13960_MOESM7_ESM.zip › Source_Data/FigureS2/FigureS2B_MotifScanOutput/KmeansCluster7/homerResults/motif25.similar.html]

motif25

## Information for motif25

G
A
T
C
G
T
A
C
A
G
T
C
A
G
T
C
G
A
T
C
G
C
A
T
A
C
G
T
C
A
T
G
A
G
T
C
G
C
A
T
A
T
C
G
A
G
C
T
A
G
C
T
A
G
T
C
  
Reverse Opposite:  

T
C
A
G
T
C
G
A
T
C
G
A
T
A
G
C
C
G
T
A
T
C
A
G
G
T
A
C
T
G
C
A
C
G
T
A
C
T
A
G
T
C
A
G
C
T
A
G
C
A
T
G
C
T
A
G
  

|  |  |
| --- | --- |
| p-value: | 1e-50 |
| log p-value: | -1.151e+02 |
| Information Content per bp: | 1.647 |
| Number of Target Sequences with motif | 102.0 |
| Percentage of Target Sequences with motif | 3.18% |
| Number of Background Sequences with motif | 157.3 |
| Percentage of Background Sequences with motif | 0.36% |
| Average Position of motif in Targets | 109.3 +/- 58.5bp |
| Average Position of motif in Background | 97.3 +/- 73.7bp |
| Strand Bias (log2 ratio + to - strand density) | -0.2 |
| Multiplicity (# of sites on avg that occur together) | 1.00 |
| Motif File: | file (matrix) reverse opposite |

### Similar de novo motifs found

|  |  |  |  |  |  |  |  |
| --- | --- | --- | --- | --- | --- | --- | --- |
| Rank | Match Score | Redundant Motif | P-value | log P-value | % of Targets | % of Background | Motif file |
| 1 | 0.803 | A T G C C G T A A C T G A G T C C G T A C T A G C T A G T C A G C T A G C A T G | 1e-26 | -60.239822 | 5.36% | 2.00% | motif file (matrix) |
